# Clade-specific ophthalmic manifestations of mpox: an umbrella review and meta-analysis of systematic reviews

**DOI:** 10.1136/bjo-2025-328294

**Published:** 2025-12-30

**Authors:** Oscar Kallay, Luxi Ji, Olalekan A Uthman, Edward J Mills, Alimuddin Zumla, Placide Mbala-Kingebeni, Jean-Jacques Muyembe-Tamfum, Elie Motulsky, Jean B Nachega

**Affiliations:** 1 Ophthalmology Department, Centre Médical de l’Alliance, Braine-l’Alleud, Walloon Brabant (Waals-Brabant), Belgium; 2 Centre Médical de l’Alliance, Brussels, Braine-I’Alleud, Belgium; 3 Division of Health Sciences, Warwick Medical School, Coventry, England, UK; 4 Department of Health Research Methods, Evidence and Impact, McMaster University, Hamilton, Ontario, Canada; 5 Division of Infection and Immunity, University College London, London, UK; 6 University College London Hospitals NHS Foundation Trust, London, UK; 7 National Institute of Biomedical Research, Kinshasa, Congo (Democratic Republic of the); 8 Department of Ophthalmology, Erasmus Hospital, Brussels, Belgium; 9 Epidemiology, Infectious Diseases and Microbiology, University of Pittsburgh, Pittsburgh, Pennsylvania, USA; 10 Epidemiology and International Health, Johns Hopkins Bloomberg School of Public Health, Baltimore, Maryland, USA; 11 Biomedical Research Institute (BMRI), Stellenbosch University Faculty of Medicine and Health Sciences (FMHS), Cape Town, South Africa

**Keywords:** Infection, Conjunctiva, Cornea, Eye (Globe), Vision

## Abstract

**Background:**

On 14 August 2024, the WHO redesignated Monkeypox (mpox) as a Public Health Emergency of International Concern. Ocular disease is under-recognised and can cause irreversible vision loss. Clarifying its burden, clade-specific patterns and HIV-related risks is essential for clinical care and public health.

**Methods:**

We conducted an umbrella review and meta-analysis, systematically searching PubMed, Embase, Scopus, Web of Science and the Cochrane Library from inception to September 2025. Eligible studies were systematic reviews reporting ophthalmic manifestations in laboratory-confirmed mpox cases, stratified by viral clades I, II and IIb. Pooled proportions were estimated using random-effects meta-analysis, with clade-stratified subgroup analyses. The protocol was registered in PROSPERO (CRD420251137180).

**Results:**

Five systematic reviews (36 primary studies; 28 139 patients) were included. The pooled proportion of conjunctivitis was 8.9% (95% CI 4.8 to 13.9), highest in clade I (21.9%) and lowest in clade IIb (2.7%). Keratitis (1.5%), eyelid lesions (3.3%), conjunctival lesions (8.4%) and corneal ulceration (3.3%) varied by clade. Visual impairment occurred in 4.5% overall; unilateral (0.9%) and bilateral (0.4%) blindness were reported only with clade I. Across reviews, people living with advanced HIV experienced disproportionately higher rates of severe and persistent ocular complications than HIV-negative individuals.

**Conclusions:**

Mpox-related ophthalmic disease can cause substantial—and potentially preventable—visual disability. Clade I infection is associated with a greater burden of ocular complications than clade II—particularly IIb. Improved access to eye health services, early recognition and integration of HIV and mpox care are critical to reducing vision loss, especially in resource-limited settings.

**WHAT IS ALREADY KNOWN ON THIS TOPIC**

- Ophthalmic complications of Monkeypox (mpox) have been described, but their overall burden, clade-specific differences and interactions with comorbidities, such as HIV, have not been quantified across reviews.

**WHAT THIS STUDY ADDS**

- To our knowledge, this is the first umbrella review to provide pooled estimates of mpox-related ocular manifestations across 28 139 patients, showing higher proportions in clade I (Ia/Ib) than clade II (IIa/IIb), and greater severity among people with advanced HIV.

**HOW THIS STUDY MIGHT AFFECT RESEARCH, PRACTICE OR POLICY**

- Findings support enhanced ophthalmic surveillance in clade I-endemic regions, integration of HIV and mpox services to address syndemic risk and incorporation of eye health into outbreak preparedness, vaccination strategies and clinical care pathways.

## Introduction

Monkeypox (mpox) is a zoonotic disease caused by the mpox virus (MPXV), an Orthopoxvirus closely related to the variola virus, which causes smallpox.^1^ First identified in monkeys in 1958, MPXV was recognised as a human pathogen in 1970 in the Democratic Republic of the Congo (DRC).^2^ Two distinct clades have been described: clade I (formerly the Congo Basin clade, subdivided into Ia and Ib) and clade II, further divided into IIa and IIb (formerly the West African clade).^3^ Clade I is more virulent, with an estimated case fatality ratio (CFR) of 10.6%, compared with 1%–3.6% for clade II.^4^ The 2022 global outbreak was caused by clade IIb, which had a CFR below 0.2%.^5^

As of August 2025, WHO data indicate substantial global transmission. In Africa alone, more than 43 000 confirmed cases were reported in the previous 12 months, with the DRC (22 222), Uganda (7979) and Sierra Leone (5245) the most affected; up-to-date figures are available via the WHO Global Mpox Dashboard and situation reports.^6–8^ Although mpox is primarily known for its cutaneous and systemic features—including fever, rash and lymphadenopathy—it can also affect ocular tissues, leading to substantial morbidity and visual impairment.^9^ Ocular involvement may result from direct viral invasion or immune-mediated mechanisms, ranging from mild conjunctivitis and blepharitis to keratitis, which can cause corneal scarring and vision loss if untreated, in patients with, or who have had, mpox.^10^ In severe cases, these complications may culminate in permanent visual disability, with major implications for patients affected, health systems and communities.^11^

Several systematic reviews have summarised evidence on mpox-related ophthalmic complications. However, no study has quantified the global burden or examined clade-specific and region-specific differences in ocular outcomes. To address this gap, we conducted an umbrella review and meta-analysis to synthesise available data on the proportion of ocular manifestations among mpox cases, with a particular focus on clade-specific variation and the influence of healthcare infrastructure.

## Methods

This umbrella review was conducted according to a protocol prospectively registered in PROSPERO (CRD420251137180) and the Preferred Reporting Items for Systematic Reviews and Meta-Analyses (PRISMA) guidelines.^12 13^

### Eligibility criteria

Defined using the Population-Outcome-Study design (POS) framework:

- *Population*: patients of all ages and demographics diagnosed with mpox.
- *Outcome*: ophthalmic manifestations and complications, including but not limited to conjunctivitis, blepharitis, keratitis, corneal lesions, impaired vision and blindness.
- *Study design*: systematic reviews and meta-analyses of observational studies, case series and case reports.

Only systematic reviews published in English were included. No restrictions were placed on publication date or geography. Where a review used a systematic search but did not fulfil all criteria for a systematic review, this was noted during data extraction.

### Information sources and search strategy

We searched PubMed, Embase, Scopus, Web of Science and the Cochrane Library from inception to 11 September 2025. The strategy, developed with an information specialist, combined keywords and Medical Subject Headings related to mpox, ophthalmic manifestations and systematic reviews. The full search strategies are provided in the online supplemental material (annex 1).

### Study selection

Two independent reviewers screened all records in Covidence (Veritas Health Innovation, Melbourne, Australia) in two stages: (1) title/abstract and (2) full text against eligibility criteria. Disagreements were resolved by discussion or adjudication by a third reviewer.

### Data collection and items

A standardised extraction form was piloted. Two reviewers independently extracted study characteristics, mpox clade, ophthalmic manifestations, proportions of ocular outcomes among mpox cases and any reported clade-specific or geographic differences. Discrepancies were resolved by consensus or input from a third reviewer. Data were managed in Microsoft Excel.

### Clade classification methodology

Because many included studies did not specify the MPXV clade, we inferred the clade based on geographic location, study period and epidemiological context:

- *Clade I* (Central African clade; Ia and Ib): studies from the DRC, Central African Republic, Republic of the Congo and Sudan. Although Cameroon has historically reported both clades I and II, no Cameroon-specific ocular studies met the inclusion criteria; therefore, Cameroon was not used to assign a clade in pooled analyses.
- *Clade II* (West African clade; IIa and IIb): studies from Nigeria and other West African countries primarily correspond to IIa. Subtype IIb was assumed for post2022 studies conducted in non-endemic settings, aligning with the multicountry outbreak beginning in 2022.

### Risk of bias assessment

We appraised the methodological quality of included reviews using AMSTAR 2 (A MeaSurement Tool to Assess Systematic Reviews).^14^ Two reviewers assessed each study independently; discrepancies were resolved by consensus.

### Synthesis of results

We calculated pooled proportions of ocular manifestations using random-effects meta-analysis (Freeman-Tukey double arcsine transformation). Subgroup analyses were conducted by clade. Given high heterogeneity (*I*²>50% in several analyses), we also reported 95% prediction intervals to reflect between-study variability.

### Terminology

‘Proportion’ refers to the frequency (%) of ocular manifestations among mpox cases in the included studies. Where no timeframe was specified, estimates represent proportion at presentation or during follow-up.

## Results

### Characteristics of the included reviews

Searches identified 71 records (online supplemental figure 1: PRISMA flow diagram). After removing 23 duplicates, 48 titles/abstracts were screened; 9 full texts were assessed, and 4 were excluded as narrative reviews, leaving 5 eligible systematic reviews (2023–2025; online supplemental table 1). These synthesised 5–25 observational studies each (published 1980–2024). Primary study quality ranged from poor to high, with most studies rated as moderate to high.

### Risk of bias assessment

AMSTAR 2 ratings are reported in online supplemental table 2.^14^ All five systematic reviews were rated as high quality, indicating generally robust methodology.

### Sample characteristics of included primary studies

36 studies reporting ocular manifestations in 28 139 patients with mpox were analysed (online supplemental table 3). The studies demonstrated substantial geographic diversity, with data from the USA (n=8), DRC (n=9), Nigeria (n=4), Mexico (n=3), Brazil (n=2), UK (n=2), Spain (n=1), France (n=1), Portugal (n=1), Canada (n=1), Saudi Arabia (n=1), Sudan (n=1), Republic of the Congo (n=1) and multi-country studies (n=2). 23 studies (2022–2024) predominantly represented clade IIb infections from non-endemic regions, while 13 earlier studies documented endemic African outbreaks associated with clade I (n=10) and clade IIa (n=3). No Cameroon-specific ocular studies were identified despite its unique epidemiological position reporting both clades. Several studies highlighted a disproportionate burden of severe and persistent ocular complications among people living with advanced HIV, particularly in clade I settings.^15 16^

### Ophthalmic manifestations by MPXV clade

#### Conjunctivitis by clade

Across 25 studies, including 27 022 patients, the pooled proportion of conjunctivitis was 8.86% (95% CI 4.84 to 13.85; prediction interval 0.00% to 43.49%) (figure 1). Clade-specific estimates differed: clade I 21.86% (95% CI 14.74 to 29.87; prediction interval 3.31% to 49.31%; *I*²=82%), clade IIa 16.91% (95% CI 12.54 to 21.74; prediction interval 9.18%–26.26%) and clade IIb 2.74% (95% CI 0.79 to 5.70; prediction interval 0.00% to 20.86%; *I*²=97.6%). Wide prediction intervals reflect expected variability across settings and study designs.

**Figure 1:**
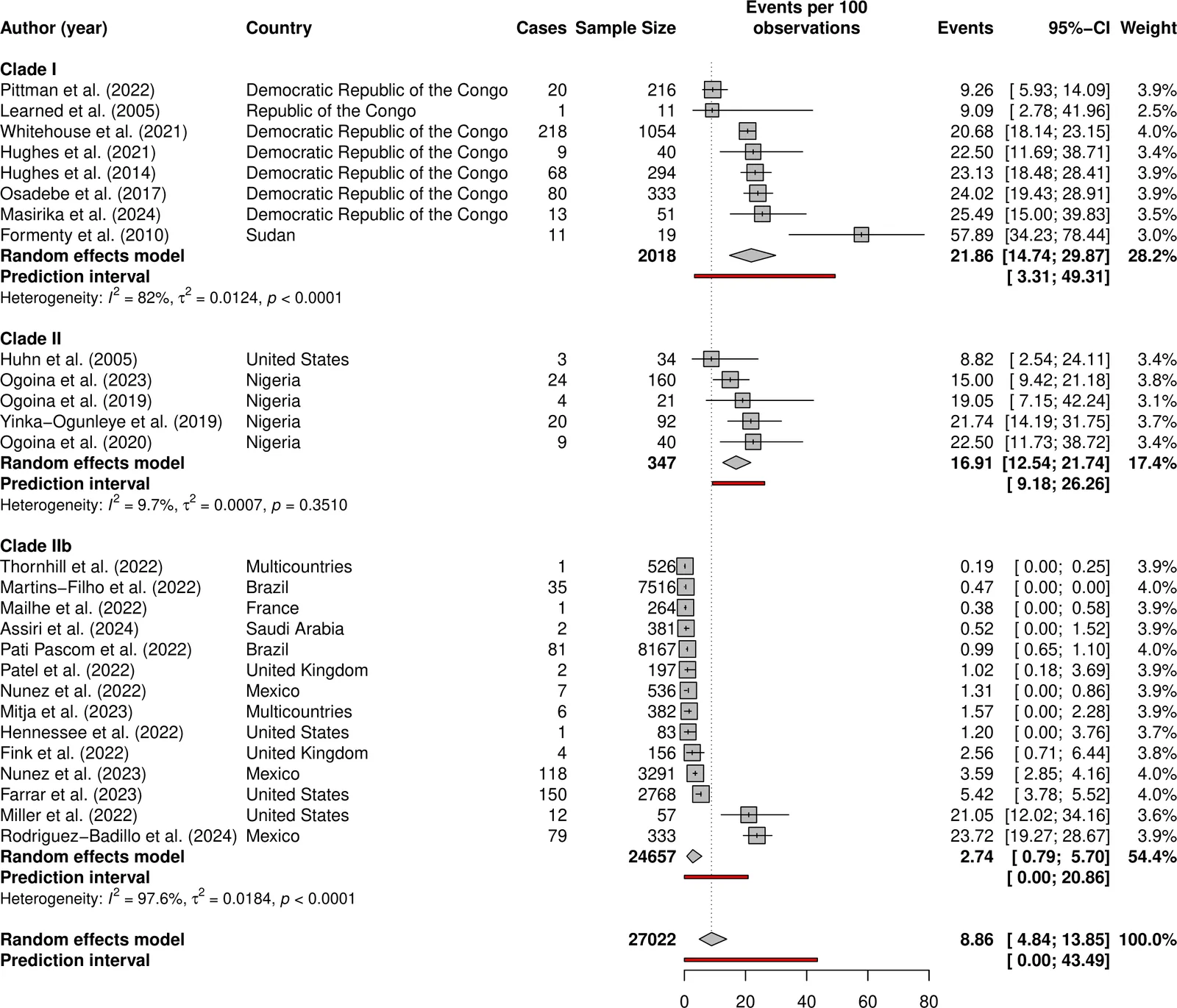
Pooled proportion of conjunctivitis by clade type.

#### Keratitis by clade

From eight studies (1957 patients), the pooled proportion of keratitis was 1.53% (95% CI 0.75 to 2.55; prediction interval 0.00% to 4.92%) (figure 2). Estimates were higher in clade I at 2.88% (95% CI 1.43 to 4.77; prediction interval 0.00% to 11.29%) than in clade IIa at 0.82% (95% CI 0.31 to 1.51; prediction interval 0.16% to 1.84%).

**Figure 2:**
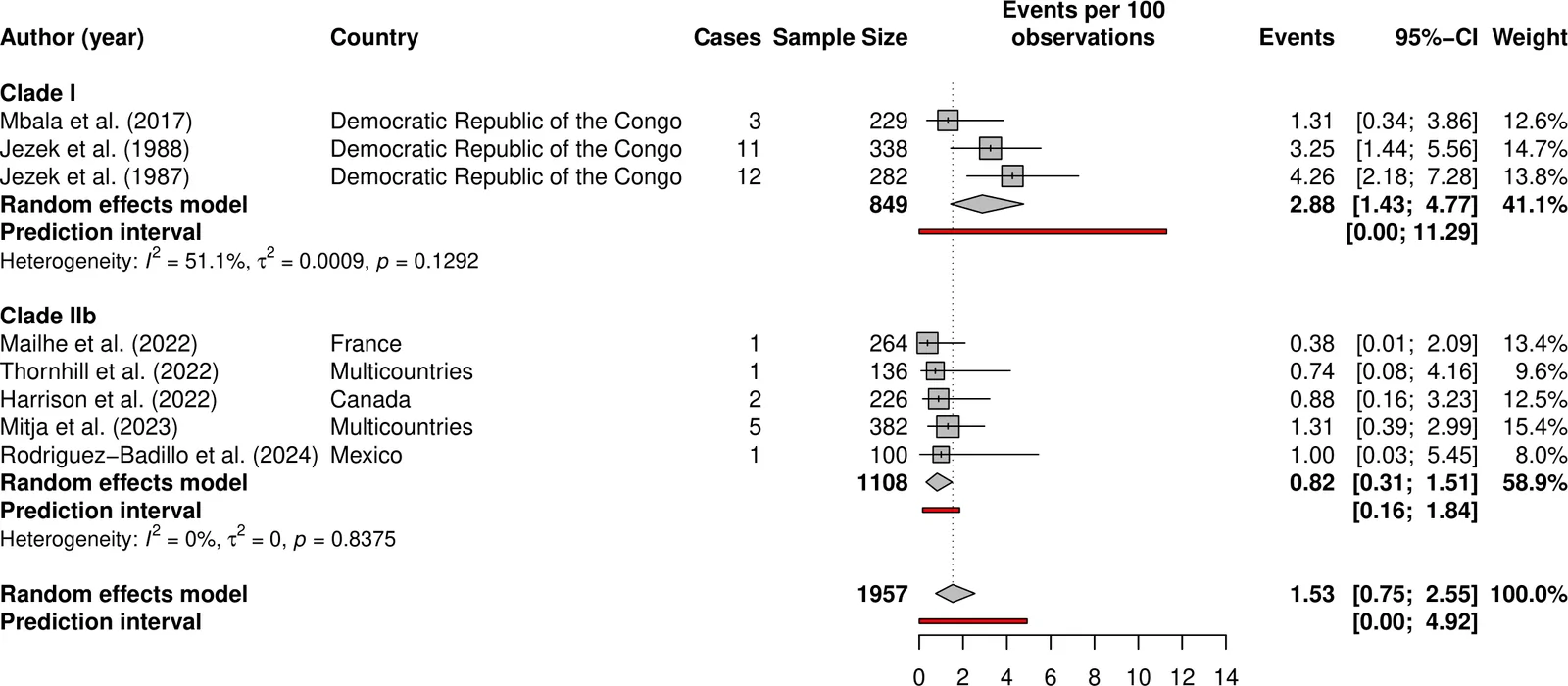
Pooled proportion of keratitis by clade type.

#### Eyelid lesions by clade

Across six studies (816 patients), the pooled proportion of eyelid lesions was 3.25% (95% CI 0.26 to 8.61; prediction interval 0.00% to 27.74%) (figure 3). Clade-specific evidence was limited: clade I 4.26% (95% CI 0.83 to 14.85; single study) and clade IIb 1.11% (95% CI 0.00 to 3.45; single study). An overall pooled estimate labelled ‘clade II’ at 3.94% (95% CI 0.00 to 13.47; prediction interval 0.00% to 52.38%) reflects mixed or unspecified II subtypes; interpret with caution.

**Figure 3:**
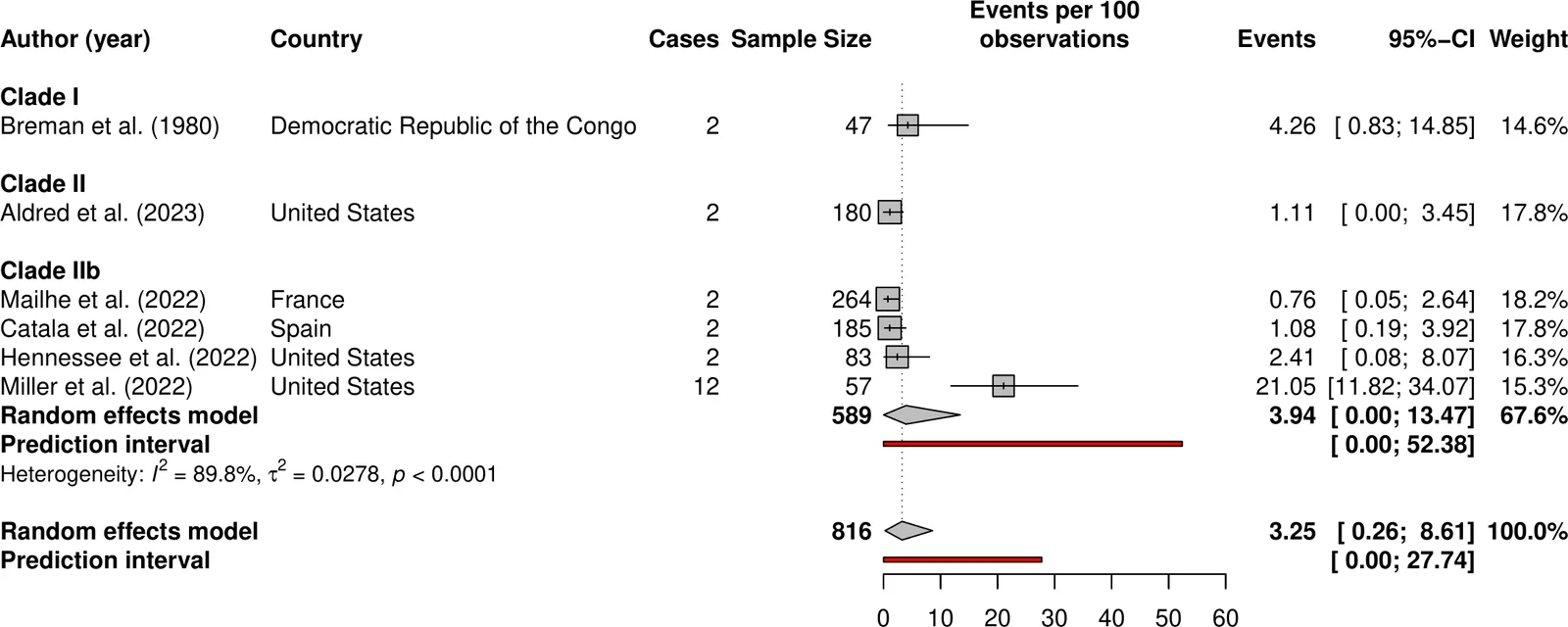
Pooled proportion of eyelid lesions by clade type.

#### Conjunctival lesions by clade

Across three studies (601 patients), the pooled proportion was 8.37% (95% CI 1.81 to 18.64; prediction interval 0.00% to 64.12%) (figure 4). Clade I was 11.61% (95% CI 2.99 to 24.70; prediction interval 0.00% to 100.00%; *I*²=93.8%; two studies), while clade II (single study) was 2.13% (95% CI 0.58 to 11.97).

**Figure 4:**
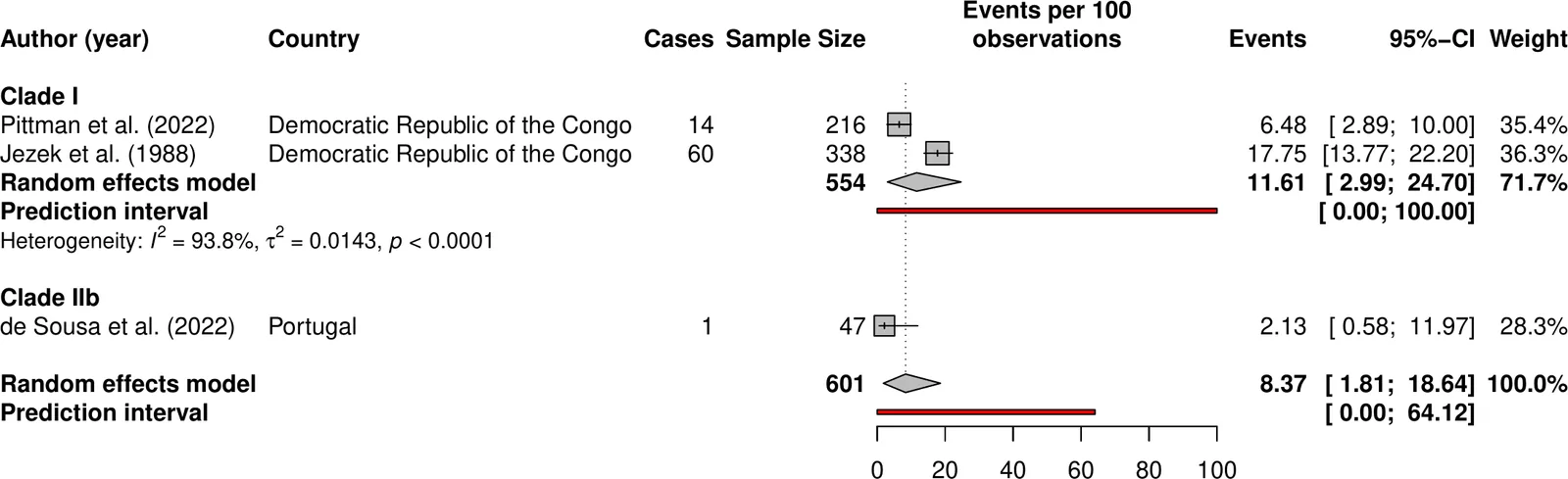
Pooled proportion of conjunctival lesions by clade type.

#### Corneal ulceration by clade

Across three studies (654 patients), the pooled proportion was 3.33% (95% CI 1.99 to 4.95; prediction interval 0.77–7.23%) (figure 5). Clade I was 3.69% (95% CI 2.32 to 5.35; prediction interval 0.00–18.99%; *I*²=0%) with consistent rates between studies (3.25% and 4.26%), and clade II (single study) was 2.94% (95% CI 0.85 to 16.19).

**Figure 5:**
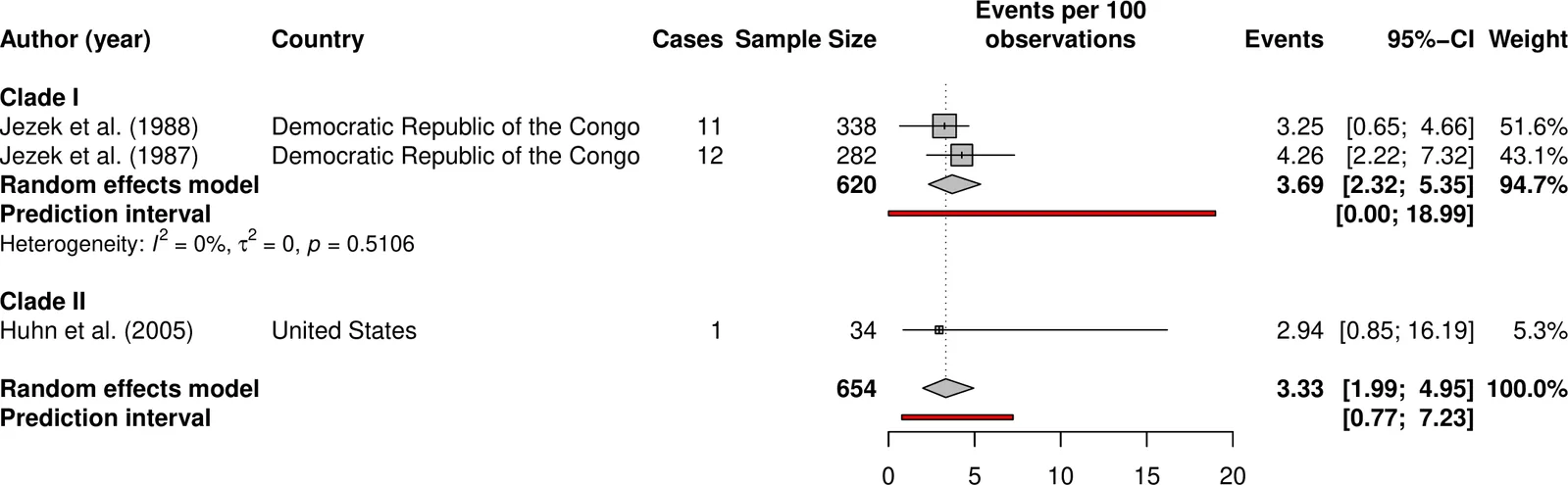
Pooled proportion of corneal ulceration by clade type.

#### Visual impairment and blindness by clade

Across five studies (986 patients; online supplemental figure 2), the pooled proportion of impaired vision was 4.48% (95% CI 1.30 to 9.21; prediction interval 0.00% to 24.37%). Clade-specific rates varied: clade I 4.20% (95% CI 1.16 to 8.88; prediction interval 0.00% to 32.30%; *I*²=87.8%), clade II 14.00% (95% CI 6.23 to 26.96; single study) and clade IIb 1.00% (95% CI 0.00 to 5.03; single study). Pooled unilateral blindness from two studies (329 patients; online supplemental figure 3) was 0.90% (95% CI 0.04 to 2.46; prediction interval 0.00% to 20.16%), both in clade I. Bilateral blindness (one clade I study; 282 patients; online supplemental figure 4) was 0.35% (95% CI 0.01 to 1.96). Subgroup analyses and case reports indicate more severe, less responsive ocular disease among people with advanced HIV, including prolonged keratitis and vision loss.^15 16^

## Discussion

This umbrella review and meta-analysis assesses the proportion of mpox-related ophthalmic manifestations, with emphasis on variation by viral clade. As noted in the Introduction, mpox comprises two primary clades that differ in clinical severity.^3 17^ Our clade-stratified analyses show consistently higher proportions of ocular complications with clade I than with clade II, and within clade II, the 2022 outbreak lineage (clade IIb) is associated with milder ocular outcomes than clade IIa.^5^

Across studies, the most frequent manifestations were conjunctivitis, conjunctival lesions, eyelid lesions and keratitis. Conjunctivitis was most common (pooled proportion 8.86%; 95% CI 4.84 to 13.85). Keratitis, eyelid and conjunctival lesions, corneal ulceration, impaired vision and blindness were less frequent but clinically meaningful. Together, these patterns support prioritising ophthalmic surveillance and early referral—particularly in settings where clade I predominates.

Differences may reflect viral and host factors (eg, tissue tropism, immune responses) and exposure patterns (eg, site of inoculation).^18–22^ Severe local inflammation, tissue necrosis and viral replication have been documented particularly in patients who are immunocompromised, which may amplify these effects.^3^ In the 2022 clade IIb outbreak driven by sexual transmission, facial involvement appears less common than in paediatric clade I cases from endemic regions; clade IIb lesions have predominantly affected genital and perianal sites, potentially reducing ocular involvement.^5^

A key limitation is the paucity of long-term ophthalmic follow-up. Most studies focused on acute manifestations, with limited information on persistence, recurrence or sequelae, such as permanent vision loss. Prognosis may differ by clade, highlighting the need for prospective, standardised ophthalmic assessments with extended patient follow-up.^10^

Beyond the meta-analysed outcomes, rare but serious complications—including periorbital cellulitis,^23^ keratouveitis,^24 25^ retinitis,^26^ endophthalmitis,^26^ sclerokeratitis,^26^ blepharoconjunctivitis^27^ and anterior uveitis^27^—have been described. Clinicians should maintain vigilance for these conditions, especially in patients with severe systemic disease, advanced immunosuppression or delayed presentation. Prompt ophthalmology referral and multidisciplinary care are critical to preventing irreversible vision loss.

### Implications for clinical and public health policy and research

Given the appreciable proportions of conjunctivitis and other ocular complications, eye health should be embedded in mpox preparedness and response through training of frontline workers, assured availability of essential ophthalmic medicines and strengthened rapid referral pathways.^28 29^ In clade I–endemic settings, clinicians should anticipate more severe ocular disease. Clinical platform data indicate an approximately sevenfold higher frequency of ocular symptoms among people living with HIV than among HIV-negative individuals, underscoring immunocompromise as a key risk factor. The mpox-HIV syndemic in Africa compounds this risk; integrated services addressing both infections are essential.^15 16^ Observational data suggest that smallpox-derived vaccines (eg, MVA-BN, LC16m8) may be associated with lower proportions of ocular complications; pre-exposure vaccination of at-risk groups and rapid postexposure vaccination of contacts should be prioritised. Paediatric evidence remains sparse despite the high paediatric burden in clade I outbreaks; prospective, standardised studies are needed to define natural history, clade-specific risks and patient-level predictors (eg, age, immune status, secondary infections).^29^

Current WHO surveillance data provide context: routine reporting captures conjunctivitis in 0.6% of cases, while patients who were hospitalised show 4.1% ocular symptoms on admission and 0.6% at discharge.^30^ These observations align with our meta-analysis and illustrate how reporting systems and patient populations influence observed frequencies.

### Study strengths and limitations

Strengths include a broad, multi-database search (plus grey literature), inclusion of historic and recent outbreaks and adherence to PRISMA and AMSTAR 2 criteria. Limitations include substantial heterogeneity, scarcity of paediatric-specific and clade-specific data, under-representation of some regions and reliance on indirect clade classification in some analyses. Cross-sectional designs predominated, limiting causal inference and long-term assessment. Despite the high paediatric case burden in clade I outbreaks (often up to 70% under 15 years), most studies did not stratify ocular outcomes by age, leaving a critical gap. Similarly, we could not systematically assess other potential risk factors (eg, degree of immunosuppression, secondary bacterial infection, nutrition, healthcare access or maternal–fetal transmission) owing to non-standardised reporting.

## Conclusions

This umbrella review synthesises current evidence on mpox-related ocular complications, identifying conjunctivitis as the most frequent manifestation. Across clades, clade I shows consistently higher proportions of ocular involvement than clade II, with clade IIb demonstrating the lowest observed burden relative to clade IIa. These findings underscore the need to embed eye health within mpox preparedness and response, including early recognition and referral, integrated HIV-mpox services and assured access to vaccines and antivirals. Priorities should focus on children and immunocompromised populations in resource-limited settings, and future research should address clade-specific risks, long-term sequelae and interactions with comorbidities such as HIV.

## Supplementary material

10.1136/bjo-2025-328294 online supplemental file 1

## Data Availability

Data are available upon reasonable request.
