# Supplementary material for: Clade-specific ophthalmic manifestations of mpox: an umbrella review and meta-analysis of systematic reviews
Source: Br J Ophthalmol. 2025 Dec 30;110(11):e328294. doi: 10.1136/bjo-2025-328294 (PMC13691674; doi:10.1136/bjo-2025-328294)
Supplement: Supplementary file 1 [file bjo-110-11-s001.pdf]

Supplementary Materials

Supplementary Table 1: Characteristics of included systematic reviews

Author	Publication Year	Databases Searched and Date	Type of Studies	Number of Studies	Year of Included Studies	Location	Meta-Analysis Performed	Quality Assessment Tool Used	Overall Quality of Studies	Outcomes of Concern
Abdelaal et al.[1]	2023	PubMed, Scopus, Web of Science, EMBASE, and Google Scholar (On August 7, 2022)	Case series, Surveillance-based study, cross-sectional, Prospective observational, Chart reviews, Retrospective observational study	11	During 2022, Before 2022	UK, Spain, DRC, US, Zaire, Nigeria, England, 16 countries, Switzerland	Yes	NIH	Average-fair	Ophthalmic manifestations
Gandhi et al.[2]	2023	PubMed, Scopus, Web of Science, EMBASE, ProQuest, EBSCOHost, Cochrane, medRxiv, arXiv, bioRxiv, BioRN, ChiRxiv, ChiRN, and SSRN (12 December 2022)	Case series, cross sectional, retrospective and prospective studies	12	1987 – 2022	Spain, USA, UK, Nigeria, DRC, Australia, Israel, Portugal, Europe	Yes	NHLBI	Fair to good	Opthalmic manifestations
Rojas-Carabali et al.[3]	2023	PubMed, Embase, VHL, MedxRiv (2023)	Meta-analysis, cross-sectional, case-control	15	2000 – 2022	Global	Yes	CLARITY, Hoy, ROBINS-I	Moderate	Uveitis, scleritis, conjunctivitis
Chenchula et al.[4]	2023	Pubmed, Google scholar, Cochrane, grey literature (May 2002 to February 2023)	Cross-sectional, retrospective	21	2021 – 2023	India, Nigeria, USA	No	Not reported	Not reported	Ocular outcomes of monkeypox
Rohilla et al.[5]	2025	PubMed, Embase, Web of Science, Scopus	Clinical trials, cross-sectional studies, cohort studies, and case series	25	1980 – 2024	United States, Democratic Republic of the Congo, Nigeria, Mexico, Brazil, United Kingdom/England, Saudi Arabia, France, Spain	Yes	MINORS (Methodological Index for Non-Randomized Studies)	Moderate	Ophthalmic manifestations

References

1. Abdelaal A, Reda A, Hassan AR, et al. Monkeypox-Associated Manifestations and Complications Involving the Eye: A Systematic Review and Meta-Analysis of Previous and Current Outbreaks. *Asia Pac J Ophthalmol (Phila)* 2023; **12**(3): 326-37.
2. Gandhi AP, Gupta PC, Padhi BK, et al. Ophthalmic Manifestations of the Monkeypox Virus: A Systematic Review and Meta-Analysis. *Pathogens* 2023; **12**(3): 452.
3. Rojas-Carabali W, Cifuentes-Gonzalez C, Agrawal R, de-la-Torre A. Spectrum of ophthalmic manifestations in monkeypox virus infection worldwide: Systematic review and meta-analysis. *Heliyon* 2023; **9**(8): e18561.
4. Chenchula S, Ghanta MK, Amerneni KC, et al. A systematic review to identify novel clinical characteristics of monkeypox virus infection and therapeutic and preventive strategies to combat the virus. *Arch Virol* 2023; **168**(7): 195.
5. Rohilla S, Gaidhane S, Balaraman AK, et al. Ophthalmic Manifestations of the Mpox Virus: A Systematic Review and Meta-analysis. *J Infect Dis* 2025; **232**(1): 220-9.

Supplementary Table 2: Risk of bias of included systematic reviews

Review Name	Protocol Registration	Comprehensive Literature Search	Risk of Bias Assessment	Publication Bias	Independent Reviewers	Data Extraction Process	Data Analysis Quality	Meta-Analysis Quality	Certainty of Evidence	Funding Transparency	Overall Quality Assessment
Abdelaal-2023[1]	Yes - PROSPERO	Yes - PubMed, Scopus, Web of Science	Yes - ROBINS-I tool	Yes - Funnel plot	Yes	Yes	Good	Random effects model	Moderate	Reported	High
Gandhi-2023[2]	Yes - PROSPERO	Extensive across multiple databases	Yes - NHLBI	Yes - Doi Plot	Yes	Yes	High	Random effects model, sensitivity analyses	High	Reported	High
Rojas-Carabali-2023[3]	Yes - PROSPERO	Thorough - PubMed, Embase, VHL, MedxRiv	Yes - CLARITY Group	Yes - Funnel plot	Yes	Yes	Good	Included (Random effects)	Moderate	Reported	High
Chenchula 2023[4]	No	Comprehensive but no gray literature	Yes - ROBINS-I	Yes	Yes	Yes	Good	Included (Random effects)	Moderate	Reported	High
Rohilla et al.[5]	Yes - PROSPERO	Yes (minor language limitation) Searched PubMed, Embase, Web of Science, and Scopus	MINORS for non-randomized studies	Yes - using Doi plot and LFK index	Yes	Yes	Yes	Included (Random effects)	Moderate	Reported	High

Supplementary Table 3: Characteristics of included primary studies

First Author	Year	Sample Size	Eye Complication	Country	Clade
Hennessee et al.[1]	2022	83	Eyelid lesions	United States	Clade IIb
Miller et al.[2]	2022	57	Eyelid lesions	United States	Clade IIb
Catala et al.[3]	2022	185	Eyelid lesions	Spain	Clade IIb
Mailhe et al.[4]	2022	264	Eyelid lesions	France	Clade IIb
Breman et al.[5]	1980	47	Eyelid lesions	Democratic Republic of the Congo	Clade I
Farrar et al.[6]	2023	2768	Conjunctivitis	United States	Clade IIb
MitjÃ et al.[7]	2023	382	Conjunctivitis	Multicountries	Clade IIb
Patel et al.[8]	2022	197	Conjunctivitis	United Kingdom	Clade IIb
Thornhill et al.[9]	2022	526	Conjunctivitis	Multicountries	Clade IIb
Nunez et al.[10]	2022	536	Conjunctivitis	Mexico	Clade IIb
Fink et al.[11]	2022	156	Conjunctivitis	United Kingdom	Clade IIb
Pascom et al.[12]	2022	8167	Conjunctivitis	Brazil	Clade IIb
Pittman et al.[13]	2022	216	Conjunctivitis	Democratic Republic of the Congo	Clade I
Martins-Filho et al.[14]	2022	7516	Conjunctivitis	Brazil	Clade IIb
Whitehouse et al.[15]	2021	1054	Conjunctivitis	Democratic Republic of the Congo	Clade I
Hughes et al.[16]	2022	40	Conjunctivitis	Democratic Republic of the Congo	Clade I
Ogoina et al.[17]	2020	40	Conjunctivitis	Nigeria	Clade II
Ogoina et al.[18]	2019	21	Conjunctivitis	Nigeria	Clade II
Yinka-Ogunleye et al.[19]	2019	92	Conjunctivitis	Nigeria	Clade II

Osadebe et al.[20]	2017	333	Conjunctivitis	Democratic Republic of the Congo	Clade I
Hughes et al.[21]	2014	294	Conjunctivitis	Democratic Republic of the Congo	Clade I
Formenty et al.[22]	2010	19	Conjunctivitis	Sudan	Clade I
Huhn et al.[23]	2005	34	Conjunctivitis	United States	Clade II
Learned et al.[24]	2005	11	Conjunctivitis	Republic of the Congo	Clade I
de Sousa et al.[25]	2022	47	Conjunctival lesions	Portugal	Clade IIb
Jezek et al.[26]	1988	338	Conjunctival lesions	Democratic Republic of the Congo	Clade I
Harrison et al.[27]	2022	226	Keratitis	Canada	Clade IIb
Mbala et al.[28]	2017	229	Keratitis	Democratic Republic of the Congo	Clade I
Jezek et al.[29]	1987	282	Keratitis	Democratic Republic of the Congo	Clade I
Ogoina et al.[30]	2023	160	Conjunctivitis	Nigeria	Clade II
Rodriguez-Badillo et al.[31]	2024	333	Conjunctivitis	Mexico	Clade IIb
Masirika et al.[32]	2024	51	Conjunctivitis	Democratic Republic of the Congo	Clade I
Nunez et al.[33]	2023	3291	Conjunctivitis	Mexico	Clade IIb
Assiri et al.[34]	2024	381	Conjunctivitis	Saudi Arabia	Clade IIb
Aldred et al.[35]	2023	180	Eyelid lesions	United States	Clade II
Zhu et al.[35]	2023	50	Impaired vision	United States	Clade II

References

1. Hennessee I, Shelus V, McArdle CE, et al. Epidemiologic and Clinical Features of Children and Adolescents Aged <18 Years with Monkeypox - United States, May 17-September 24, 2022. *MMWR Morb Mortal Wkly Rep* 2022; **71**(44): 1407-11.
2. Miller MJ, Cash-Goldwasser S, Marx GE, et al. Severe Monkeypox in Hospitalized Patients - United States, August 10-October 10, 2022. *MMWR Morb Mortal Wkly Rep* 2022; **71**(44): 1412-7.

3. Catala A, Clavo-Escribano P, Riera-Monroig J, et al. Monkeypox outbreak in Spain: clinical and epidemiological findings in a prospective cross-sectional study of 185 cases. *Br J Dermatol* 2022; **187**(5): 765-72.
4. Mailhe M, Beaumont AL, Thy M, et al. Clinical characteristics of ambulatory and hospitalized patients with monkeypox virus infection: an observational cohort study. *Clin Microbiol Infect* 2023; **29**(2): 233-9.
5. Breman JG, Kalisa R, Steniowski MV, Zanotto E, Gromyko AI, Arita I. Human monkeypox, 1970-79. *Bull World Health Organ* 1980; **58**(2): 165-82.
6. Farrar JL, Lewis NM, Houck K, et al. Demographic and Clinical Characteristics of Mpox in Persons Who Had Previously Received 1 Dose of JYNNEOS Vaccine and in Unvaccinated Persons - 29 U.S. Jurisdictions, May 22-September 3, 2022. *Am J Transplant* 2023; **23**(2): 298-303.
7. Mitja O, Ogoina D, Titanji BK, et al. Monkeypox. *Lancet* 2023; **401**(10370): 60-74.
8. Patel A, Bilinska J, Tam JCH, et al. Clinical features and novel presentations of human monkeypox in a central London centre during the 2022 outbreak: descriptive case series. *BMJ* 2022; **378**: e072410.
9. Thornhill JP, Barkati S, Walmsley S, et al. Monkeypox Virus Infection in Humans across 16 Countries - April-June 2022. *N Engl J Med* 2022; **387**(8): 679-91.
10. Nunez I, Garcia-Grimshaw M, Ceballos-Liceaga SE, et al. Epidemiological and clinical characteristics of patients with human monkeypox infection in Mexico: a nationwide observational study. *Lancet Reg Health Am* 2023; **17**: 100392.
11. Fink DL, Callaby H, Luintel A, et al. Clinical features and management of individuals admitted to hospital with monkeypox and associated complications across the UK: a retrospective cohort study. *Lancet Infect Dis* 2023; **23**(5): 589-97.
12. Pascom ARP, Souza IN, Krummenauer A, et al. Epidemiological and clinical characteristics of monkeypox cases in Brazil in 2022: a cross-sectional study. *Epidemiol Serv Saude* 2022; **31**(3): e2022851.
13. Pittman PR, Martin JW, Kingebeni PM, et al. Clinical characterization of human monkeypox infections in the Democratic Republic of the Congo. *medRxiv* 2022: 2022.05.26.22273379.
14. Martins-Filho PR, Nicolino RR, da Silva K. Incidence, geographic distribution, clinical characteristics, and socioeconomic and demographic determinants of monkeypox in Brazil: A nationwide population-based ecological study. *Travel Med Infect Dis* 2023; **52**: 102517.
15. Whitehouse ER, Bonwitt J, Hughes CM, et al. Clinical and Epidemiological Findings from Enhanced Monkeypox Surveillance in Tshuapa Province, Democratic Republic of the Congo During 2011-2015. *J Infect Dis* 2021; **223**(11): 1870-8.
16. Hughes CM, Liu L, Davidson WB, et al. A Tale of Two Viruses: Coinfections of Monkeypox and Varicella Zoster Virus in the Democratic Republic of Congo. *Am J Trop Med Hyg* 2020; **104**(2): 604-11.
17. Ogoina D, Iroezindu M, James HI, et al. Clinical Course and Outcome of Human Monkeypox in Nigeria. *Clin Infect Dis* 2020; **71**(8): e210-e4.
18. Ogoina D, Izibewule JH, Ogunleye A, et al. The 2017 human monkeypox outbreak in Nigeria-Report of outbreak experience and response in the Niger Delta University Teaching Hospital, Bayelsa State, Nigeria. *PLoS One* 2019; **14**(4): e0214229.
19. Yinka-Ogunleye A, Aruna O, Dalhat M, et al. Outbreak of human monkeypox in Nigeria in 2017-18: a clinical and epidemiological report. *Lancet Infect Dis* 2019; **19**(8): 872-9.
20. Osadebe L, Hughes CM, Shongo Lushima R, et al. Enhancing case definitions for surveillance of human monkeypox in the Democratic Republic of Congo. *PLoS Negl Trop Dis* 2017; **11**(9): e0005857.
21. Hughes C, McCollum A, Pukuta E, et al. Ocular complications associated with acute monkeypox virus infection, DRC. *International Journal of Infectious Diseases* 2014; **21**: 276-7.

22. Formenty P, Muntasir MO, Damon I, et al. Human monkeypox outbreak caused by novel virus belonging to Congo Basin clade, Sudan, 2005. *Emerg Infect Dis* 2010; **16**(10): 1539-45.
23. Huhn GD, Bauer AM, Yorita K, et al. Clinical characteristics of human monkeypox, and risk factors for severe disease. *Clin Infect Dis* 2005; **41**(12): 1742-51.
24. Learned LA, Reynolds MG, Wassa DW, et al. Extended interhuman transmission of monkeypox in a hospital community in the Republic of the Congo, 2003. *Am J Trop Med Hyg* 2005; **73**(2): 428-34.
25. de Sousa D, Patrocinio J, Frade J, et al. Monkeypox Diagnosis by Cutaneous and Mucosal Findings. *Infect Dis Rep* 2022; **14**(5): 759-64.
26. Jezek Z, Grab B, Szczeniowski M, Paluku KM, Mutombo M. Clinico-epidemiological features of monkeypox patients with an animal or human source of infection. *Bull World Health Organ* 1988; **66**(4): 459-64.
27. Harrison LB, Bergeron G, Cadieux G, et al. Monkeypox in Montreal: Epidemiology, Phylogenomics, and Public Health Response to a Large North American Outbreak. *Ann Intern Med* 2023; **176**(1): 67-76.
28. Mbala KP, Huggins WJ, Riu RT. The clinical characterization of human monkeypox infections in the Democratic Republic of Congo. *Trop Med Int Health* 2017; **22**: 31.
29. Jezek Z, Szczeniowski M, Paluku KM, Mutombo M. Human monkeypox: clinical features of 282 patients. *J Infect Dis* 1987; **156**(2): 293-8.
30. Ogoina D, Dalhat MM, Denue BA, et al. Clinical characteristics and predictors of human mpox outcome during the 2022 outbreak in Nigeria: a cohort study. *Lancet Infect Dis* 2023; **23**(12): 1418-28.
31. Rodriguez-Badillo P, Rodriguez-Aldama JC, Gabian-Fortes LDC, et al. Mpox-Related Ophthalmic Disease: A Retrospective Observational Study in a Single Center in Mexico. *J Infect Dis* 2024; **229**(Supplement_2): S255-S9.
32. Masirika LM, Udahemuka JC, Ndishimye P, et al. Epidemiology, clinical characteristics, and transmission patterns of a novel Mpox (Monkeypox) outbreak in eastern Democratic Republic of the Congo (DRC): an observational, cross-sectional cohort study. *medRxiv* 2024: 2024.03.05.24303395.
33. Nunez I, Ceballos-Liceaga SE, de la Torre A, et al. Predictors of laboratory-confirmed mpox in people with mpox-like illness. *Clin Microbiol Infect* 2023; **29**(12): 1567-72.
34. Assiri AM, Alserehi H, Abuhasan MY, et al. Epidemiology, clinical presentation, and outcome of mpox: A study of 381 cases in Saudi Arabia. *IJID Reg* 2024; **11**: 100358.
35. Aldred B, Kelley C, Colasanti J. Characteristics of the 2022 mpox outbreak in a southeastern US city. *Topics in Antiviral Medicine* 2023; **31**.

Supplementary Figure 1: PRISMA Flow for studies identification

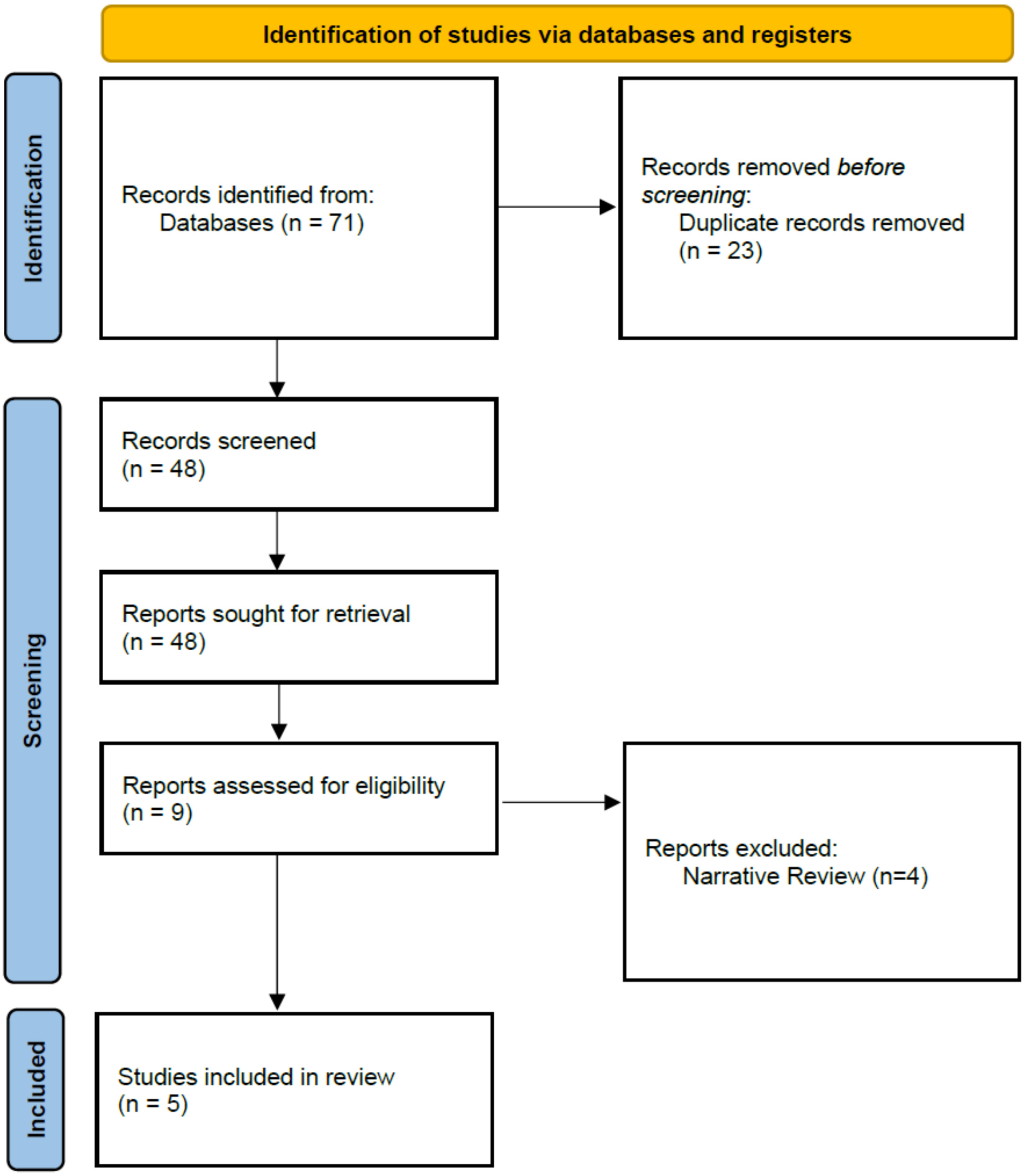

Supplementary Figure 2: Pooled prevalence of impaired vision by Clade Type

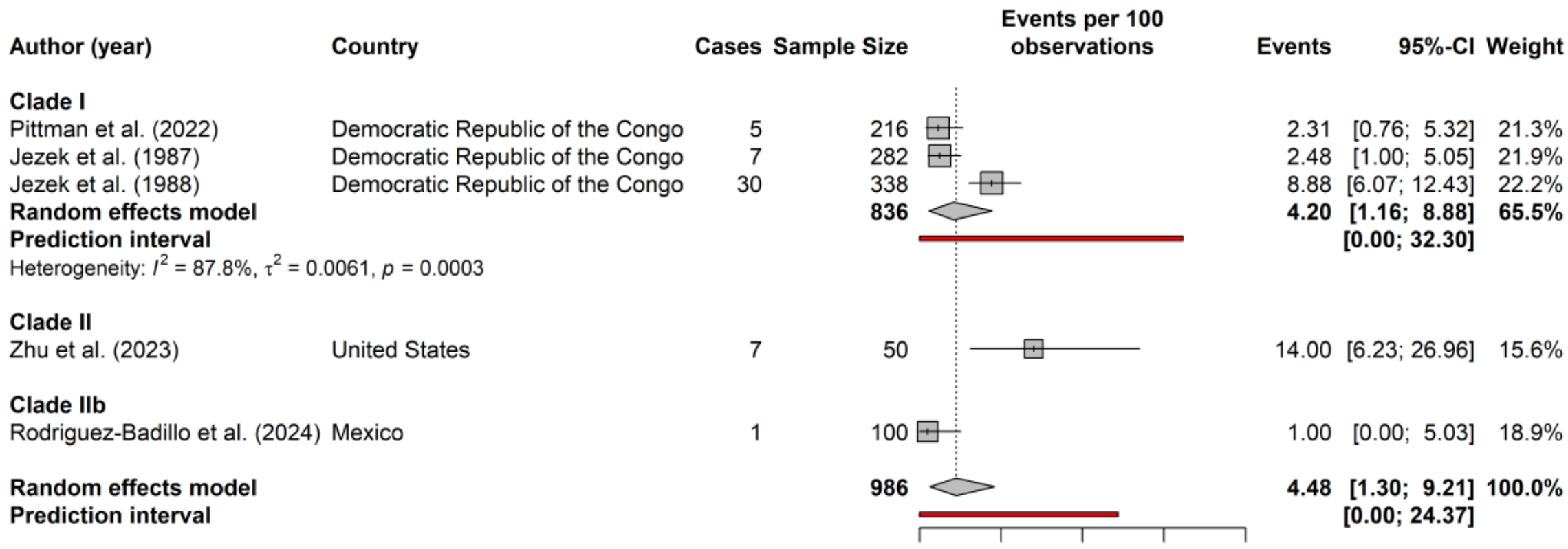

Supplementary Figure 3: Pooled prevalence of unilateral blindness by Clade Type

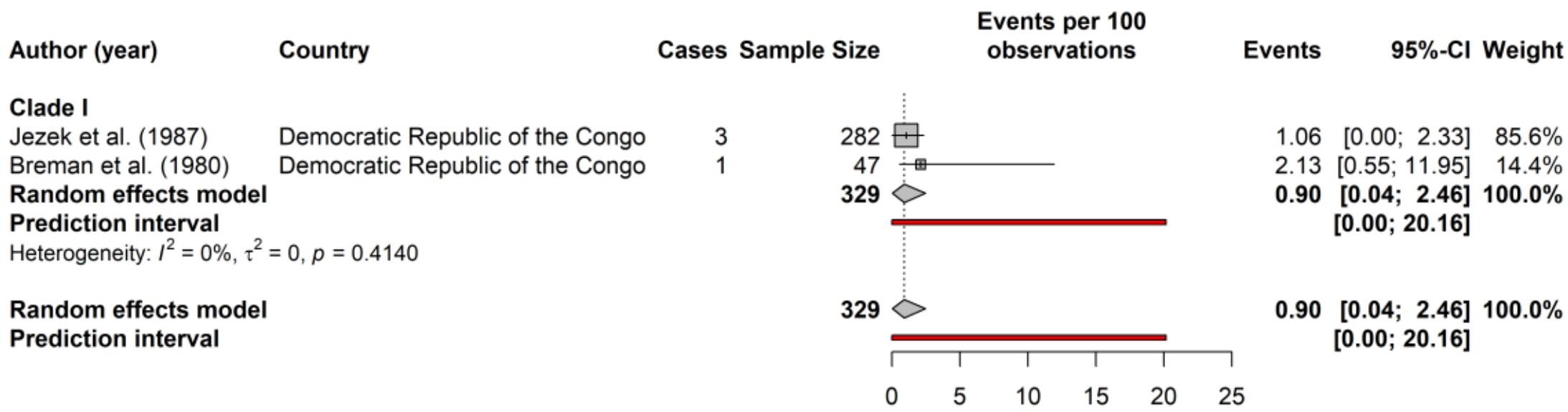

Supplementary Figure 4: Pooled prevalence of bilateral blindness by Clade Type

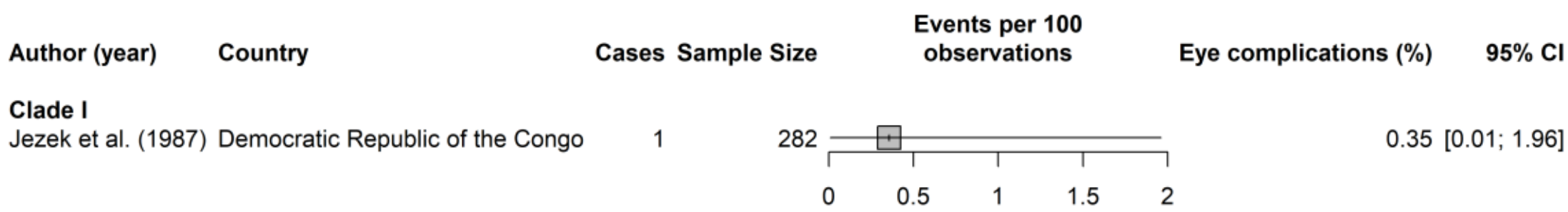

Annex 1: Search Strategy

Database: Ovid MEDLINE(R) and Epub Ahead of Print, In-Process, In-Data-Review & Other Non-Indexed Citations, Daily and Versions
Date run: September 1, 2025
Years: Database inception–present
Language: No limits

Block 1 — Mpox / Monkeypox

1. exp Mpox/
2. exp Monkeypox/
3. exp Mpox virus/
4. exp Monkeypox virus/
5. (mpox or “monkey pox” or monkeypox or MPXV or (“mpox virus” or “monkeypox virus”)).ti,ab,kw.
6. orthopoxvirus.ti,ab,kw.
7. (orthopox adj3 (simian or primate or monkey)).ti,ab,kw.
8. (variola virus adj3 (simian or primate or monkey)).ti,ab,kw.
9. 1 or 2 or 3 or 4 or 5 or 6 or 7 or 8

Block 2 — Ophthalmic / Eye

10. exp Eye Diseases/
11. exp Eye/
12. exp Vision Disorders/
13. exp Conjunctivitis/ or exp Keratitis/ or exp Corneal Diseases/
14. exp Eyelid Diseases/ or exp Blepharitis/
15. exp Uveitis/ or exp Retinitis/ or exp Endophthalmitis/
16. exp Orbit/ or exp Orbital Diseases/
17. (ophthalm or ocular or eye or eyes).ti,ab,kw.
18. (conjunctiv or keratit or cornea).ti,ab,kw.
19. (blepharit or eyelid or “eye lid” or “eye lids”).ti,ab,kw.
20. (uveitis or retinitis or endophthalmitis).ti,ab,kw.
21. (“visual impairment” or “vision loss” or blind).ti,ab,kw.
22. (“corneal ulcer” or “corneal lesion” or “conjunctival lesion”).ti,ab,kw.
23. (periorbital or orbital or intraocular).ti,ab,kw.
24. 10 or 11 or 12 or 13 or 14 or 15 or 16 or 17 or 18 or 19 or 20 or 21 or 22 or 23

Block 3 — Systematic review / Meta-analysis core filter

25. Meta-Analysis.pt. or Systematic Review.pt.
26. (systematic review or meta-analysis or meta analy).ti,ab,kw.
27. (“pooled analysis” or “umbrella review” or “evidence synthesis”).ti,ab,kw.
28. (literature review adj3 (systematic or comprehensive)).ti,ab,kw.
29. (cochrane or medline or pubmed or embase).ti,ab,kw.
30. ((review or overview) adj3 (systematic or quantitative)).ti,ab,kw.
31. (“search strategy” or “data extraction” or “study selection”).ti,ab,kw.
32. 25 or 26 or 27 or 28 or 29 or 30 or 31

Block 4 — Additional review signals (sensitivity layer)

33. Review.pt. and (systematic or meta).ti,ab,kw.
34. (PRISMA or PROSPERO or GRADE).ti,ab,kw.
35. (“random effects” or “fixed effects” or heterogeneity or “forest plot” or “funnel plot” or “risk of bias”).ti,ab,kw.

36. 33 or 34 or 35

Combine

37. 9 and 24 and (32 or 36)
